# The Book World of Medicine and Science

**Published:** 1899-12-30

**Authors:** 


### The Book World of Medicine and Science.

Who's Who. 1900. An Annual Biographical Dictionary. Fifty-second year of issue. (London: Adam and Charles Black. 1900. Price 3s. 6d. net).

"Who's Who" begins the new century, if we are so to consider it, better than ever, and fuller than ever of useful information. We miss on its title page the name of the editor under whose supervision the book burst into new popularity a few years ago, but otherwise there is little that is fresh to note except continued progress, and in consequence increasing usefulness. Some of the tables have been omitted, such as those headed "Commons" and "Peers," but the information which was contained in these is all to be found in the biographies; the other tables, however, have been added to. That the book fulfils a widefelt want is well proved by its success; and, indeed, one almost wonders how one ever did without it. Who is who? used often to be a serious and sometimes an unanswerable question, but by aid of "Who's Who" all is now made plain.

Cancer. By Thomas W. Nunn, F.R.C.S. (Small 8vo. Pp. 89. 11 Plates.)

This is a small general treatise on carcinoma, which does not profess to be dogmatic on any of the many important questions still open to surgeons, but touches briefly on most of them. The general impressions of a surgeon who has had such exceptional opportunities as those afforded by the Middlesex Hospital deserve respectful consideration, but we are bound to urge that it would have been better to leave some of these important questions untouched than to have dismissed them with the summary criticism that Mr. Nunn affords. Mr. Stiles' paper is referred to in four lines, without any reference as to where it is to be found (*Edinburgh Medical Journal*, 1892), while no hint is given as to the important lines of cleavage in Stiles' paper from the descriptions of the mammary lymphatics given by Heidenhain or Langhams — differences which bear strongly on the extent and direction of operative removal. Mr. Nunn leans to the "constitutional" factor in cancer. He points out (and from a surgeon of his experience the point is of great value) that in all cases of "chimney sweeps' cancer," a form of epithelioma regarded as strictly local and of irritation origin, there is invariably a co-existence of generally disseminated sessile warts. Another detail of personal observation we must recall from these pages as of exceptional worth: that small, hard masses of mammary tumour, indistinguishable by touch from ordinary adenomata, which are found in the periphery of the gland, are from their position very suspicious.

Various statistics of 1,000 consecutive cases at the Middlesex Hospital are given. The analysis of the birthplaces of patients shows a large preponderance in the South-Eastern registration district, which might merely imply that most patients in London were drawn naturally from this district, but so far as it goes, it confirms the enormous excess of deaths from cancer, furnished by the Registrar-General's returns, in this division of England. The Middlesex Hospital analysis of regional (body) incidence of cancer confirms that of other English observers, if the practice of the whole hospital, as distinguished from the cancer wards, is thrown together as it should be. All French and Swiss writers give a far higher proportion of cases of cancer of the stomach, as compared with cancer of the breast and uterus than English writers give. Of this no sufficient explanation has yet been given. We regret to find in these pages no sufficient estimate of the value of early operation, particularly no references to the large, radical operations of Halsted, and others. No attempt is made, however, in these pages to discuss any form of operative technique. The illustrations are from excellent preparations, and are well reproduced.

The Pathologist's Handbook. By T. N. Kelynack, M.D., M.R.C.P. (London: T. and A. Churchill. 1899. Price 4s. 6d. Pp. 186, 12mo.)

Dr. Kelynack has here produced a little manual of great value, though perhaps of undue brevity. Certainly Dr. Kelynack's aim is, as he says, to indicate methods; but in these days the tendency is, very rightly, to make medical works complete in themselves, and we feel inclined to call upon Dr. Kelynack, who has furnished us with such an excellent skeleton, to clothe the dry bones of this little work with living flesh. Dr. Kelynack has, however, contented himself with giving us directions and a scheme for post-mortem examination, and has done his task admirably. One little omission we note—the omission of the very excellent method of section of the lungs long used at Brompton and commended by Dr. Fowler. This method is, we believe, unrivalled for the just displaying of morbid pulmonary affections. The evils arising from too compressed a style are, however, seen on page 149. On this page, as an exemplification of Dr. Kelynack's admirable advice to have all ante-mortem wounds drawn or photographed, are given, on the authority of Dr. Ogston, two pictures representing homicidal and suicidal cutthroat. But there is no descriptive letterpress, and, as in the pictures homicidal cutthroat is represented from left to right and suicidal cutthroat from right to left, confusion is sure to occur. It appears to us that either the pictures have been transposed or that in the process of reproduction reversal has occurred, for at present the pictures represent the conditions named as seen in a mirror. There is a rather ludicrous error on page 166, where a common scoop is said to be useful for "bailing out." The inverted commas are the author's. "Bailing out" is surely a legal procedure, and "baling out" is what should be printed. It is only because Dr. Kelynack's little work, as a whole, is so excellent, so sagacious, and so well written that we are constrained to point out these unfortunate slips.

Report on Investigations by the U.S. Fish Commission in Mississippi, Louisiana, and Texas, in 1897-1899. By Barton W. Evermann. (Washington: Government Printing Office. 8vo., pp. 23. 28 Plates.)

This report should make the mouth of the naturalist or fisherman to water. There are 74 distinct species of fish catalogued as met with. One of the commonest is the "Blue cat-fish," probably Amiurus ponderosus, which attains a size of 100 lb.! The object of this report was to investigate the failing "cat-fish" industry. The term "cat-fish" is very loose; in England it connotes a destructive form of Blenny (Anarrhicas lupus, "Wolf-fish"), a ferocious creature often caught on cod-lines. In the Northern States the name covers about 16 species of the genus Pimelus, of which the Horned Pout is one of the commonest and poorest river fish in the Eastern State rivers. The "amiurus" of the Southern States is a very superior creature, as is the Buffalo, which reach 35 lb.; "gaspergon," 20 lb.; and the "goujon" (Leptops olivaris), the head alone of which has turned 20 lb., the whole fish often scaling 70 lb. No mention is made as to whether these fine fish afford sport on the rod like the Tarpon of the Western Gulf. Every zoologist interested in fish should secure this pamphlet. Many obscure points in classification are authoritatively cleared, some excellent anatomical details are given, and the plates are admirable.

